# Assessment of bioelectrical impedance analysis devices for data reliability of body impedance measurements

**DOI:** 10.3389/fnut.2025.1705346

**Published:** 2026-01-13

**Authors:** Dan Bee Kim, Seong Su Shin, Wan-Seop Kim

**Affiliations:** Korea Research Institute of Standards and Science, Daejeon, Republic of Korea

**Keywords:** bioelectrical impedance analysis, body impedance imitator, calibration, data reliability, standard

## Abstract

**Background:**

Bioelectrical impedance analysis (BIA) devices are widely used for body composition analyses, but their accuracy for body impedance measurements can vary significantly between BIA device models. Calibration of BIA device is essential to ensure the reliability of measurement data, particularly for clinical and research purposes. This study aims to evaluate the electric impedance measurement abilities of multi-frequency BIA devices using a home-built body impedance imitator (BII), designed to simulate human body impedance, and to assess the measurement uncertainty based on calibration with internationally accredited reference standards.

**Methods:**

Two different models of multi-frequency BIA device with similar specifications and measurement schemes were evaluated using the BII as a reference standard. First, the impedance values of the BII were calibrated using an LCR meter, traceable to national impedance standards. Next, the impedance measurements were performed using both models based on the standard BII over a wide frequency range (1 kHz to 1 MHz) to analyze the accuracy, linearity, contact resistance effects, etc. of the BIA devices.

**Results:**

Despite having similar specifications, two models of BIA device exhibited notable differences in their measurement performance. Model A showed better accuracy and consistency, with measurement uncertainty of approximately 1.4% at a 95% confidence level (*k* = 2). In contrast, Model B showed higher deviations, particularly in trunk impedance measurements. The study found that the BIA device calibration procedure could effectively quantify the measurement uncertainty and ensure the reliability of measurement data provided by the BIA device.

**Conclusion:**

This study demonstrates that BIA devices can be calibrated using a standard procedure with a stable reference such as the BII, which traces back to internationally accredited impedance standards. The results show that the calibration significantly improves the reliability of BIA device measurements, providing a level of confidence for the measurement data.

## 1 Introduction

So called bioelectrical impedance analysis (BIA) devices are now widely used in hospitals for medical checkups and clinical examinations, as well as in fitness centers or even at home. Since the advent of the BIA, research studies have been actively conducted in terms of instrument developments and clinical applications (1–14). As a result, the BIA technology has matured over last several decades, and its industry has expanded significantly along with the fast growing health industry.

Since a BIA device estimates the body composition, such as body cell mass, total body water, and fat-free body mass, etc., based on the electrically measured body impedance, its impedance measurement capability is fundamental in determining the instrument’s basic specifications. Thus, when assessing the accuracy of a BIA device, its impedance measurement performance should be primarily evaluated. Furthermore, as the BIA device impedance measurements can be affected by various factors such as electrode contact, environmental conditions, and inherent characteristics of the device itself, an accurate calibration process is required in order to ensure reliability of the body impedance measurement data.

Yet, previous studies on the measurement reliability assessments of BIA devices focused mostly on the final data of body composition analysis, in comparison with the other well-known methods, such as dual energy X-ray absorptiometry (15–21), and less on the raw data of body impedance. Only a few studies have been conducted to explore the accuracy of BIA devices in electrical impedance measurements including the research conducted at National Institute of Standards and Technology from a metrology perspective (22–24).

The BIA technology is increasingly recognized as a valuable clinical tool from the basic nutritional status assessment to clinical applications such as hydration monitoring in dialysis and oncology (25–29). Such expansion can be supported by studies on the reliability of BIA devices. Hence, it is essential to know how accurately a BIA device can measure an individual’s body composition. When the instrument’s measurement accuracy is evaluated based on references, which are traceable to national standards, the measurement data will obtain certified reliability, potentially with an international equivalence. So far, most BIA device manufacturers have paid little attention to such verifications since there are no strict legal requirements. However, the situation is expected to change due to the recent increase in regulatory demands. The BIA device manufacturing industry can move toward adapting the concept of standardization, which provides a reliability in terms of manufacture, safety, and data.

Our interests lie within exploring the confidence level of the electrical impedance data, measured by BIA devices. In advance, we investigated the performance of two models of BIA device on the same human body and found some notable differences in their measured impedance values despite of their similar measurement principles and claimed capabilities. The results will be presented again in detail in the next section. At this point, such distinction between the measurement data highlights the motivation of this study.

Therefore, this study aims to provide the calibration uncertainty of BIA devices in order to confirm that their measurement data can be trusted with a high level of confidence. First, basic principles of the BIA were examined to devise a method for the BIA device assessment. As a result, a body impedance imitator (BII), simulating a human body in terms of electric impedance, was developed to be employed as a reference standard in BIA device calibrations. A BIA device calibration was then conducted using the developed BII, and its calibration uncertainty was analyzed.

## 2 Methods

### 2.1 Body impedance measurement principle

A human body reacts to an electric current like an electrical impedance *Z*, which is a complex quantity, composed of resistance *R* and reactance *X*. Various kinds of body tissues respond differently to the flowing current according to their electrical properties. For instance, muscles with a higher water content experience less resistance compared to the relatively water-free tissues such as fat. The reactance, coming from the capacitance of cell membrane increases with the cell number (30). Moreover, the body impedance varies with the frequency of the current flow. At low frequencies below 1 ~ 5 kHz, extracellular fluids primarily determine the impedance. In contrast, as the frequency increases, the cell membrane capacitance decreases, and the current starts flowing through both the extracellular and the intracellular fluids (2, 9, 31, 32).

**Figure 1:**
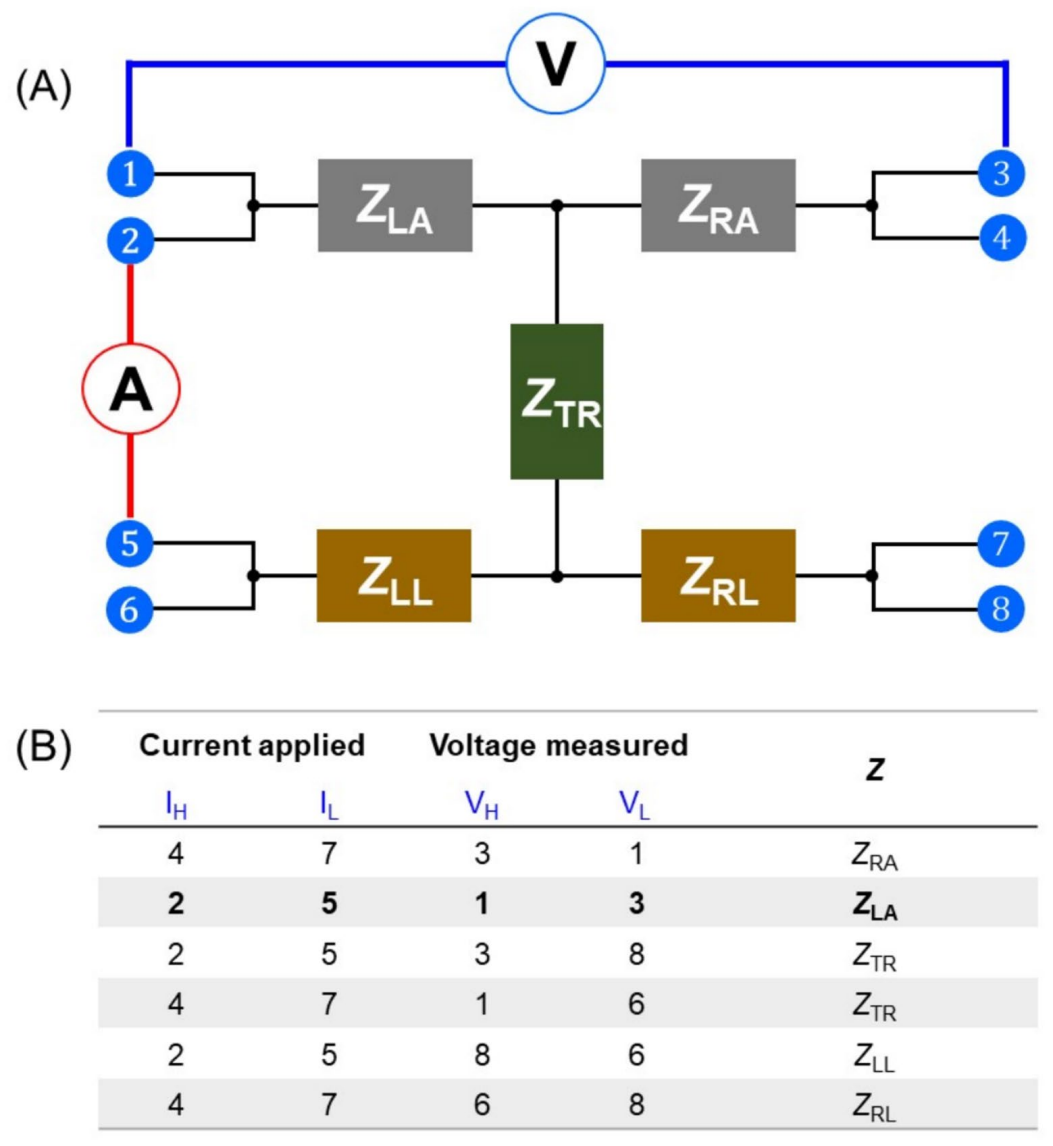
**(A)** Body impedance measurement schematic of an eight-polar bioelectrical impedance analysis device for five body parts—right arm (*Z*_RA_), left arm (*Z*_LA_), left leg (*Z*_LL_), right leg (*Z*_RL_), and trunk (*Z*_TR_)—with eight electrodes at hands and feet. **(B)** Among the eight electrodes, four are selected in sequence to measure the impedance of each body part using a 4-terminal method. For example, *Z*_LA_ can be measured by applying current from electrode 2 to 5 while measuring the voltage across electrode 1 and 3.

Based on these characteristics, the body impedance measurements across a wide frequency range enable the body composition analysis. Accordingly, body impedance models and measurement methods have been investigated to achieve optimal performance under ideal conditions. In general, high performance BIA devices measure the electrical impedance of human body using the eight-polar configuration. The basic measurement principle is illustrated in Figure 1A. The body impedance model divides the body into five parts; right arm (RA), left arm (LA), left leg (LL), right leg (RL), and trunk (TR). Each part is connected to two electrodes, either on a hand or a foot, except for the trunk, and the impedance of each part is measured in a 4-terminal way by applying current through two electrodes and measuring voltage across the other two electrodes. For instance, when measuring the impedance of LA (*Z*_LA_), a current is applied between the electrodes of 2 and 5 while voltage is measured between the electrodes of 1 and 3. Impedance of all body parts can be measured in a similar way, one after the other as described in Figure 1B.

In this work, two commercial BIA device models of A and B, both measuring the segmental impedance of five body parts based on the eight-polar configuration at multi-frequencies, were studied. The main objective of the study is to demonstrate the necessity of device calibration through comparative analysis. The BIA devices examined are the ACCUNIQ BC720 and the InBody 770, which are widely used in hospitals and health centers for professional analyses, including clinical trials. These models are produced by different manufacturers, but they basically work based on similar measurement principles and exhibit comparable specifications with measurement ranges of (10 Ω ~ 1,000 Ω) and (1 kHz ~ 1 MHz). Although they each claim similar measurement abilities for the body impedance, they have not been evaluated yet on the same basis of an accredited system. Through our study of device evaluation and calibration, the measurement performance of these two BIA devices can be compared equivalently based on a standard procedure.

**Figure 2:**
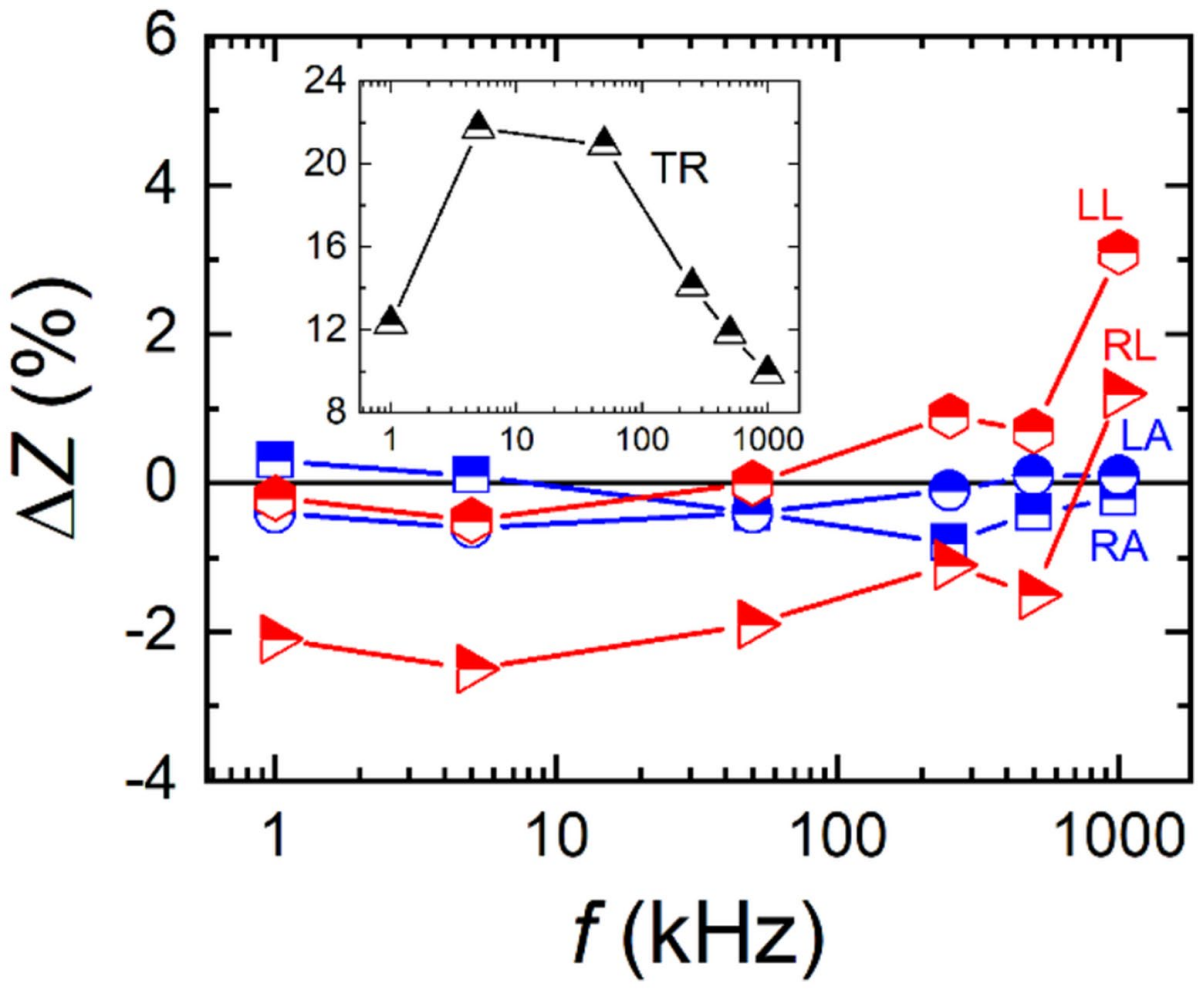
Impedance measurement results for the same human body using two different models of body impedance analysis device. Relative differences between the models are plotted against the frequency for all five body parts. The inset shows the trunk part separately.

Priorly, two models of BIA devices were employed to measure the impedance of the same human body. The measurements were made consecutively with a short time interval in order to have the human body condition as same as possible. Figure 2 presents relative differences between the measured impedance values of the two BIA devices for all five body parts against the frequency. The differences were less than 1% for both arms while they were larger for legs especially at the highest frequency of 1 MHz. The difference in TR impedance was about one order of magnitude higher, reaching up to 22% at maximum as shown in the inset of Figure 2. The larger difference in TR impedance is mainly due to the smaller impedance of TR. While the limbs have impedances in the range of several hundred ohms, the TR impedance is roughly one order of magnitude lower. Thus, 1 Ω difference in the impedance measurement corresponds to a few percentage errors for the limbs but several tens of percentage error for the TR, respectively. Such a comparison demonstrates differences in the impedance measurements between the two BIA devices.

Unfortunately, a human body is not an ideal standard reference even if the same human body is used. This is because human body is an active system, which constantly changes by many factors both in short- and long-terms. Hence, it is recommended to introduce a BII as an alternative. Unlike a human body whose impedance varies depending on body hydration level, body posture, body condition, skin moisture level of hands and feet, etc. (33–38), an imitator consisting of passive elements of resistors and capacitors would be more stable and practical in many ways.

### 2.2 Body impedance imitator development

In purpose of calibrating BIA devices using a reference, a reliable BII is necessary. Since there are currently no commercial BIIs available for sale, studies were carried out to develop one for the BIA device calibration.

**Figure 3:**
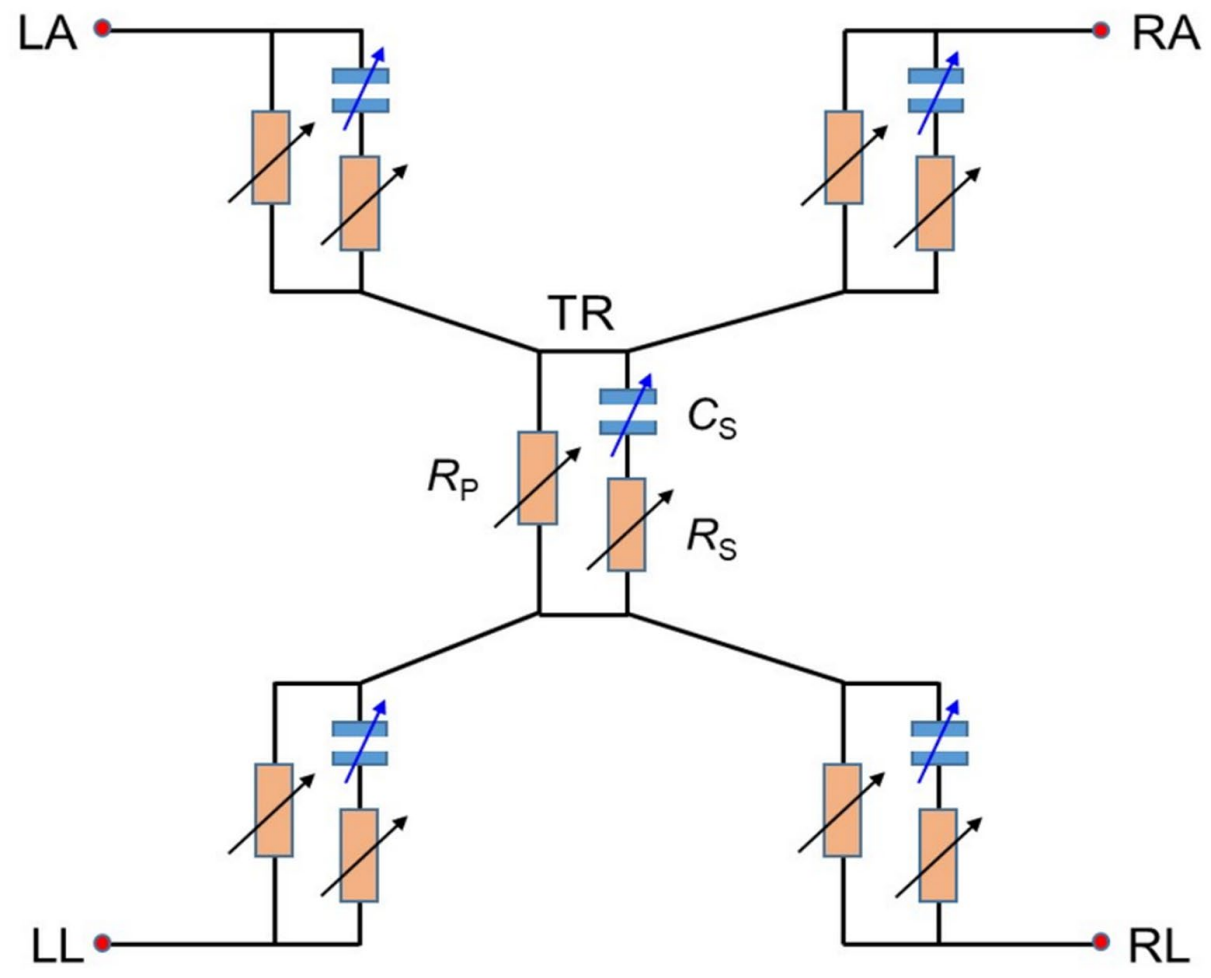
Basic circuit model of a human body impedance, divided into five parts of right arm (RA), left arm (LA), left leg (LL), right leg (RL), and trunk (TR), each consisting of a resistor (*R*_P_) in parallel, a series resistor (*R*_S_), and a series capacitor (*C*_S_).

Figure 3 shows a simple circuit model for the body impedance. As aforementioned, the body impedance can be divided into five parts of four limbs and one trunk, and each part is modeled as a homogeneous conductor, consisting of a resistor (*R*_P_) in parallel, a series resistor (*R*_S_), and a series capacitor (*C*_S_). *R*_P_ represents the extracellular fluid while *R*_S_ and *C*_S_ represent the intracellular fluid and the cell membrane, respectively (22). At low frequencies below 1 ~ 5 kHz, most current flows through the extracellular path while at high frequencies, current can pass through the cell membrane as its impedance decreases (2, 9, 31, 32).

### 2.3 Variable body impedance imitator (VBII)

**Figure 4:**
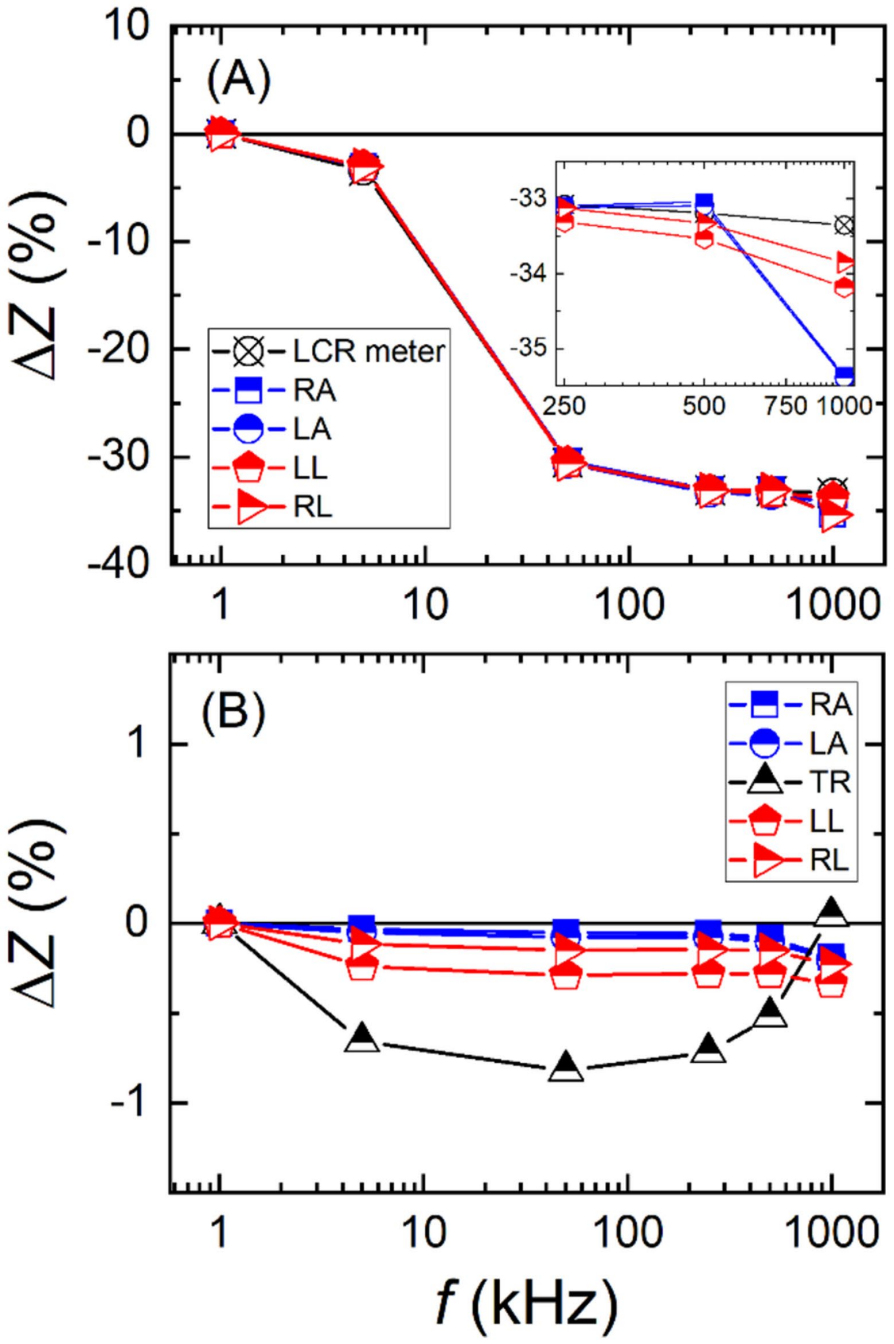
Relative changes of impedance values, measured by a body impedance analysis device, against the frequency for different body impedance imitators of **(A)** variable body impedance imitator and **(B)** programmable body impedance imitator.

At the beginning, the imitator was constructed with fixed values for each body part. Impedance of each body part was determined according to actual body impedance of an adult male. After conducting some initial tests, the imitator was upgraded to include a variable feature, allowing for more convenient evaluation of BIA devices over a wide impedance range as real human body exhibits different spectrum of body impedance according to the sex, age, pathological condition, etc. The variable body impedance imitator (VBII) was constructed with manually adjustable five channels, corresponding to five body parts. Each body part consists of *R*_P_, *R*_S_, and *C*_S_. The dynamic range of each channel was designed to be from 1 Ω to 1,000 Ω in resistance and from 1 pF to 1 μF in capacitance. Figure 4A shows impedance changes of the VBII against the frequency. The limb impedances were measured using an LCR (Inductance *L*, Capacitance *C* and Resistance *R*) meter and the BIA device model A, and they changed more than 35% at the highest frequency of 1 MHz when compared to the lowest frequency of 1 kHz as can be seen more closely in the inset. Although the impedance values measured by the BIA device model A matched well with those measured by the LCR meter, the frequency dependency of VBII was rather large. Since a real body impedance does not exhibit such a large frequency dependency, it was decided to further improve the imitator.

### 2.4 Programmable body impedance imitator (PBII)

An improved programmable body impedance imitator (PBII) was designed to have a negligible frequency dependency within the measurement range utilizing the series *RLC* resonance effect. In a series *RLC* circuit, the inductive reactance of the inductor becomes equal to the capacitive reactance of the capacitor (*X*_L_ = *X*_C_) at a specific frequency point. This results in the cancellation of the two reactances, and the impedance of the series *RLC* circuit becomes purely “real” at the resonance frequency. Then, the total impedance of the series *RLC* circuit corresponds only to the resistance, and the frequency effect becomes negligible.

As presented in Figure 4B, the frequency dependency of the PBII, measured by the BIA device model A, remained flat within 1% over the whole frequency range and for all body parts. In addition, the impedance values of the PBII can be set via a PC with a deviation less than 0.01% (TR: 2%) from the nominal value. The PBII is also designed for on-site calibration, so its physical size is compact for convenient transport.

## 3 Body impedance analysis device evaluation results

Two aforementioned representative models of BIA devices were evaluated using the developed BIIs in terms of measurement accuracy, resolution, linearity, contact resistance, etc.

### 3.1 Impedance measurement accuracy

**Figure 5:**
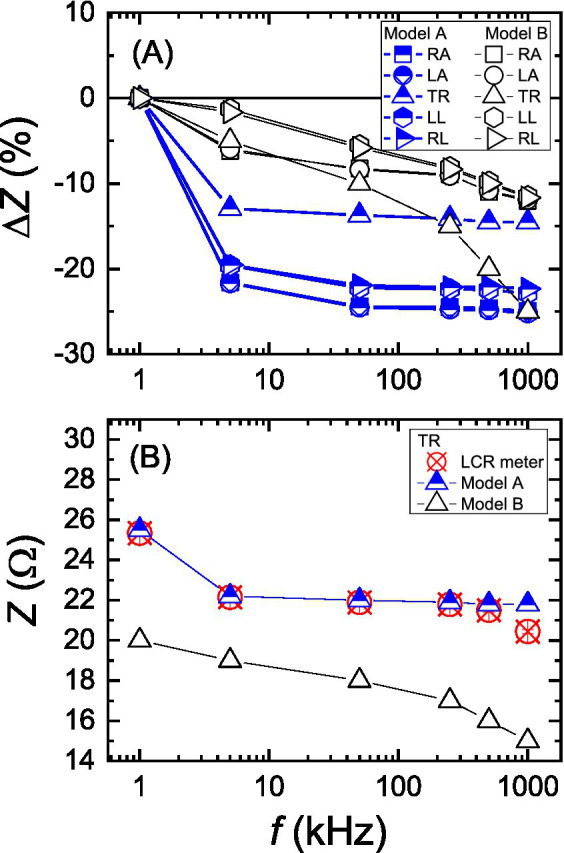
Measurement results for the same impedance imitator using two different body impedance analysis devices of model A and B. **(A)** Relative changes in the measured impedance values with respect to 1 kHz for all five body parts and **(B)** absolute values of the measured impedance for the trunk against the frequency.

First, impedance values of the BII were measured using both BIA device models of A and B. Although each BIA device measured the same imitator, two models showed distinct frequency dependencies for all body parts as can be seen in Figure 5A. Impedance measurement results in absolute values are also plotted in Figure 5B for the trunk along with LCR meter measurement results. The trunk impedance values, measured by the model A agree better with the reference impedance values, measured by an LCR meter. At 1 kHz, the measured trunk impedance value for the model B differs by about 20% from that of the model A.

**Figure 6:**
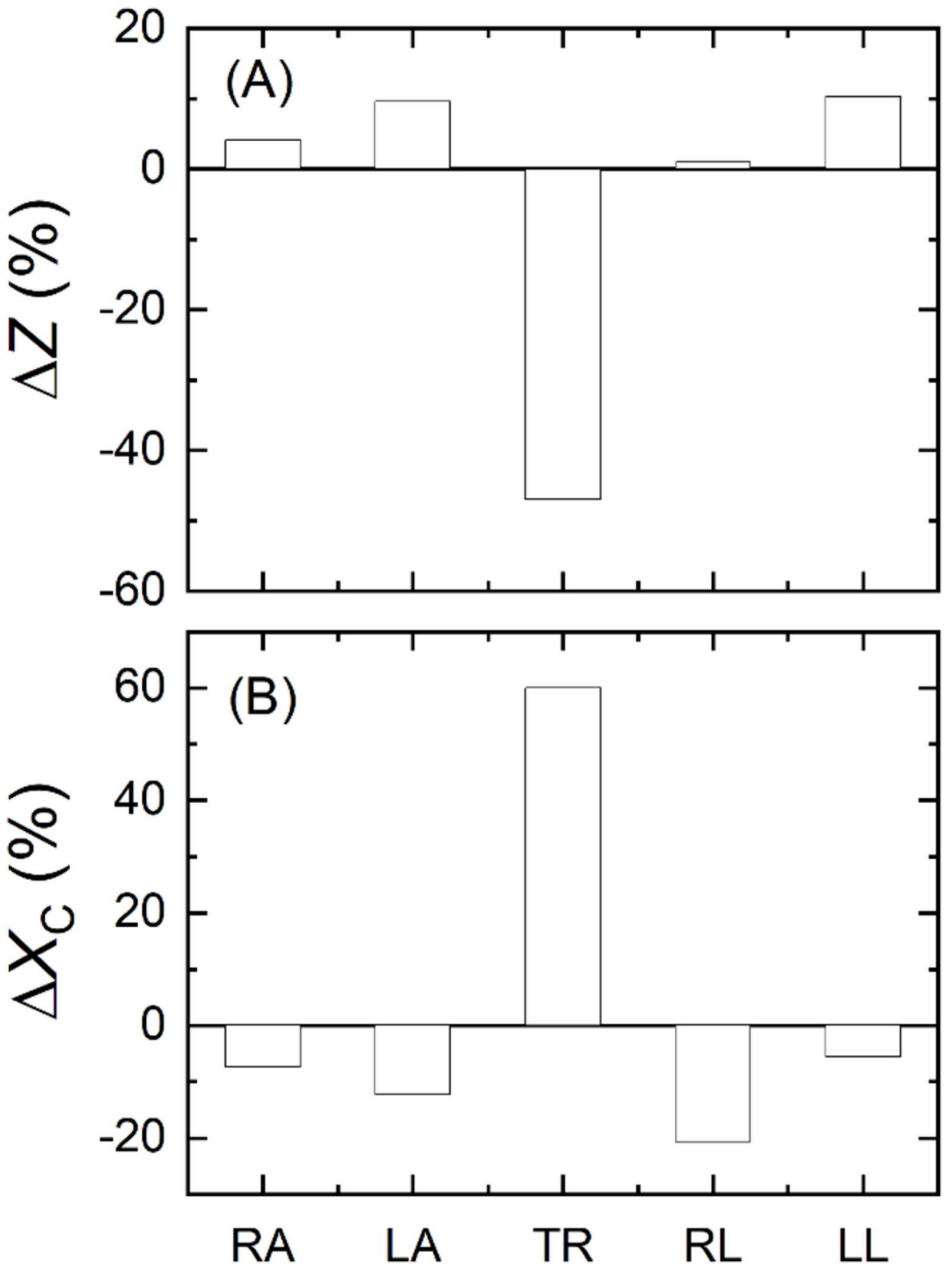
Measurement results for the same body impedance imitator using two different models of body impedance analysis devices. Relative differences between the models in **(A)** impedance and **(B)** reactance values of all five body parts at 50 kHz.

In another measurements using the VBII, differences between two BIA device models in the impedance and reactance values were noticed again as plotted in Figure 6 for all five body parts at 50 kHz. The differences were as large as 20% for the limbs, and they were more than 50% for the trunk, observed similarly in both impedance and reactance. This difference in the impedance measurements also resulted in different body composition analyses; there was about 50% difference in the calculated body water contents between the BIA device models.

**Figure 7:**
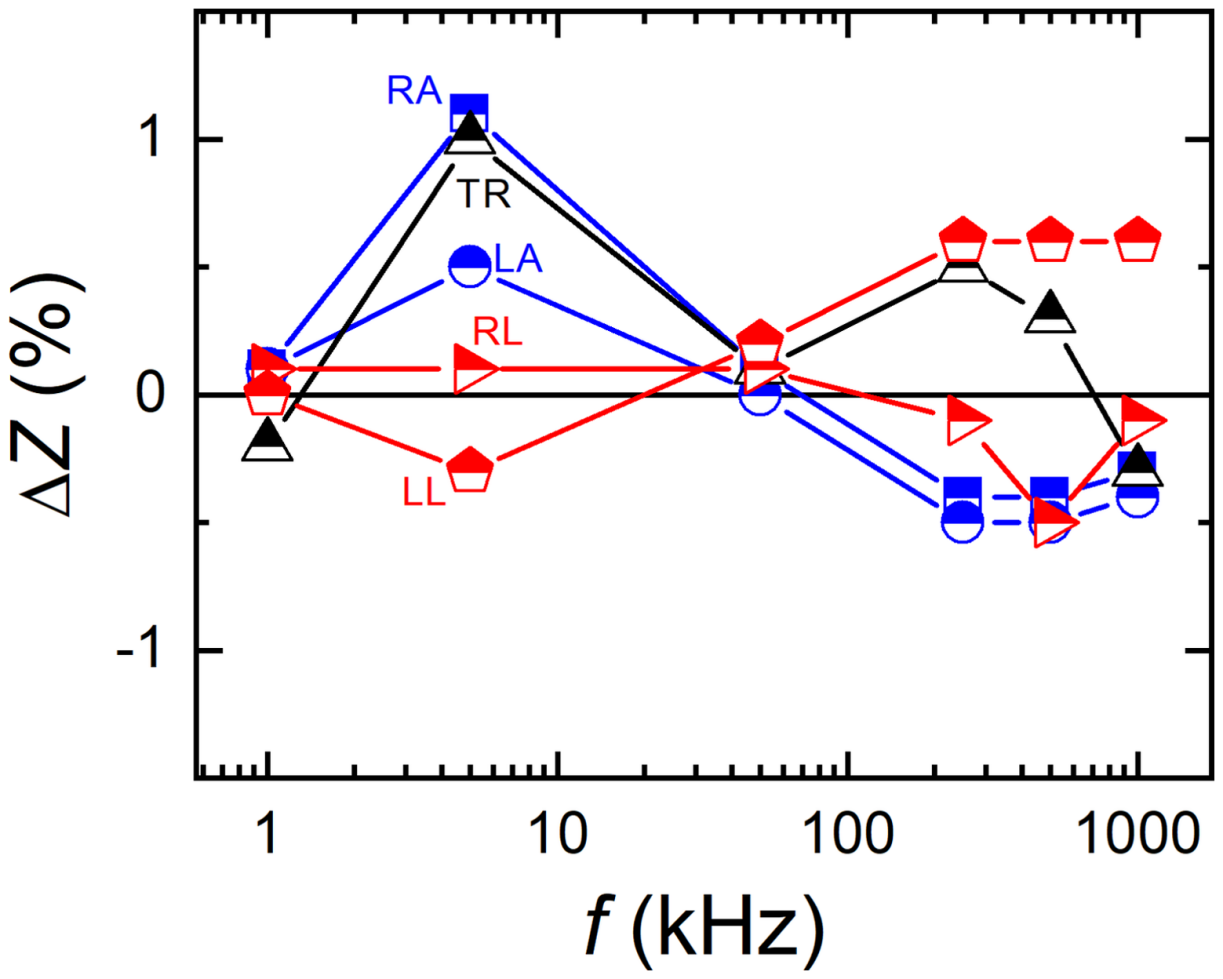
Relative differences between the LCR meter and the body impedance analysis device of model A in the measured impedance values of a body impedance imitator for all five body parts.

While measurement differences were observed between the BIA device models, it was repeatedly confirmed that the measured impedance values of the model A agreed well with those of LCR meter. Figure 7 shows that the measured impedance values between the BIA device model A and the LCR meter agreed with each other within ±1% range for all body parts. The study results indicate that BIA devices may have different measurement accuracies despite of their similar specifications, claimed by manufacturers. Indeed, the BIA device calibration can improve reliability of the measurement data.

### 3.2 Impedance measurement resolution

**Figure 8:**
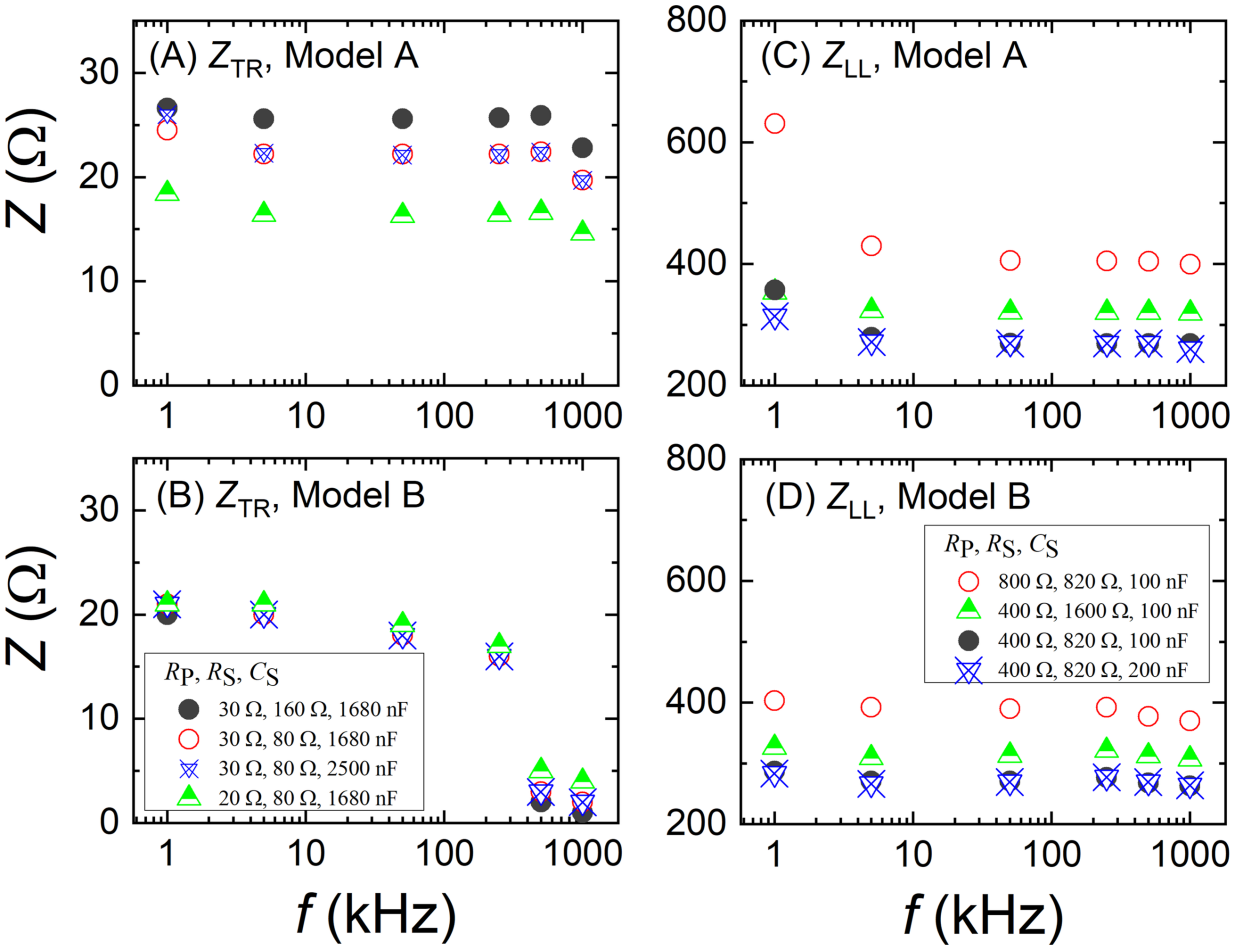
Trunk impedance (*Z*_TR_) values measured by different body impedance analysis devices of model **(A)** A and **(B)** B against the frequency. Left leg impedance (*Z*_LL_) values measured by different body impedance analysis devices of model **(C)** A and **(D)** B against the frequency. Resistor and capacitor elements of a variable body impedance imitator were adjusted to test the measurement resolution as described in the legends.

In purpose of assessing the measurement resolution of the BIA devices, the VBII was employed. As aforementioned, each body part of the imitator consists of *R*_P_, *R*_S_, and *C*_S_ elements, as illustrated in Figure 3. Their values were adjusted as shown in legends of Figure 8. Figures 8A,B present impedance values of TR, measured by the models of A and B, respectively. In addition to the distinct frequency responses between the models, it is observed that the model A distinguishes the different impedance values of the imitator set as *R*_P_ = 20 Ω and 30 Ω, whereas the model B does not. Figures 8C,D present impedance values of LL, measured by the models of A and B, respectively. Here, since the LL impedance is about one order larger than the TR impedance, the model B was able to distinguish the different impedance values set as *R*_P_ = 400 Ω and 800 Ω. Yet, the difference measured by the model B was only about 100 Ω at 1 kHz. In case of *C*_S_, the model B appears unable to discriminate a difference of 100 nF at all frequencies while the model A shows some difference at low and high frequencies. Overall, the model A seems to have a better measurement resolution by about one order of magnitude.

### 3.3 Linearity

**Figure 9:**
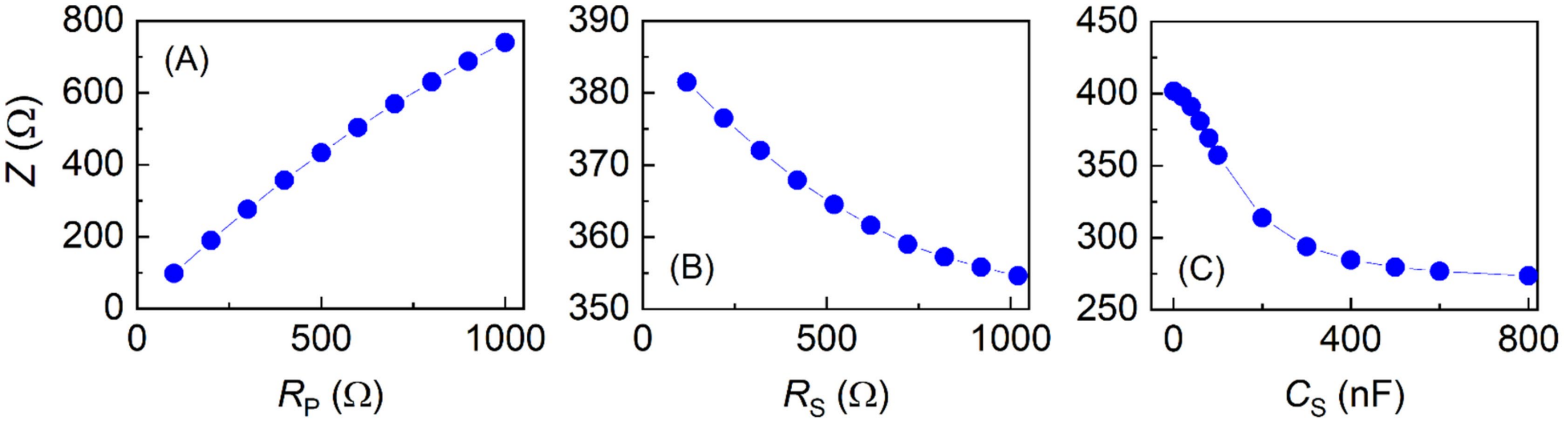
Impedance measurement results of the body impedance analysis device model A at 50 kHz by varying **(A)** parallel resistance (*R*_P_), **(B)** series resistance (*R*_S_), and **(C)** series capacitor (*C*_S_) of the variable body impedance imitator.

The VBII was further employed to evaluate the linearity of BIA devices by varying values of the elements of *R*_P_, *R*_S_, and *C*_S_. While the value of one element had been varied, the values of the other two were remain fixed at their values. The measurement results are presented in Figure 9 for the model A. When *R*_P_ was varied from 100 Ω to 1,000 Ω, the impedance increased almost linearly as shown in Figure 9A. Variations in *R*_S_ and *C*_S_ also gave expectable changes in the impedance changes as presented in Figures 9B,C.

### 3.4 Contact resistance

**Figure 10:**
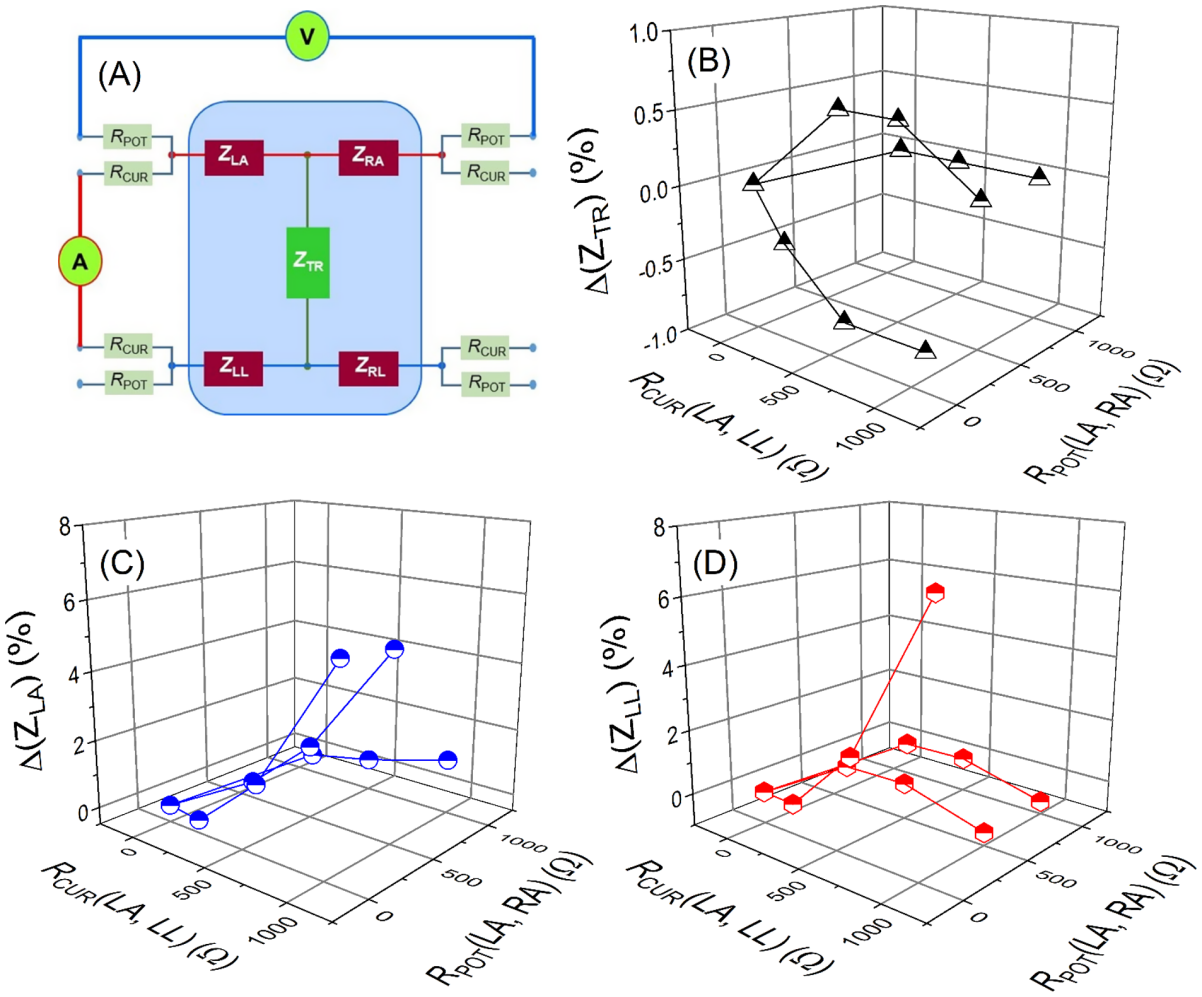
**(A)** Experimental schematic of resistors attached in series to the electrodes of the body impedance analysis device: *R*_POT_: series resistor at the potential electrode (0–1,000 Ω) and *R*_CUR_: series resistor at the current electrode (0–1,000 Ω). Relative changes of **(B)** trunk (*Z*_TR_), **(C)** left arm (*Z*_LA_), and **(D)** left leg (*Z*_LL_) impedance values with respect to the zero series (no series resistor) resistor conditions against the varying series resistances at 250 kHz.

BIA device measurements can be affected by contact resistance between the electrode and the skin of human body being measured, which is why manufacturers emphasize the importance of proper electrode contact in their instrument manuals. Hence, studies were done to explore the contact resistance effects by attaching resistors in series to the electrodes. Various combinations were measured by the BIA device model A, and one representative case is presented here. Series resistors were attached at the potential electrodes of LA and RA (*R*_POT_) and the current electrodes of LA and LL (*R*_CUR_) as illustrated in Figure 10A. The series resistances were varied from 0 to 1,000 Ω, and Figure 10 shows relative changes of (B) TR, (C) LA, and (D) LL impedance values with respect to the zero (no series resistor) series resistor condition against the varying series resistances at 250 kHz. Despite the series resistors, the impedance measurement values for TR changed by less than 1% as shown in Figure 10B and even less for RA and RL. Meanwhile, the measured impedance values of LA and LL were changed up to 5% at certain conditions. It is likely due to the fact that the series resistors were connected to the current electrodes of LA and LL.

**Figure 11:**
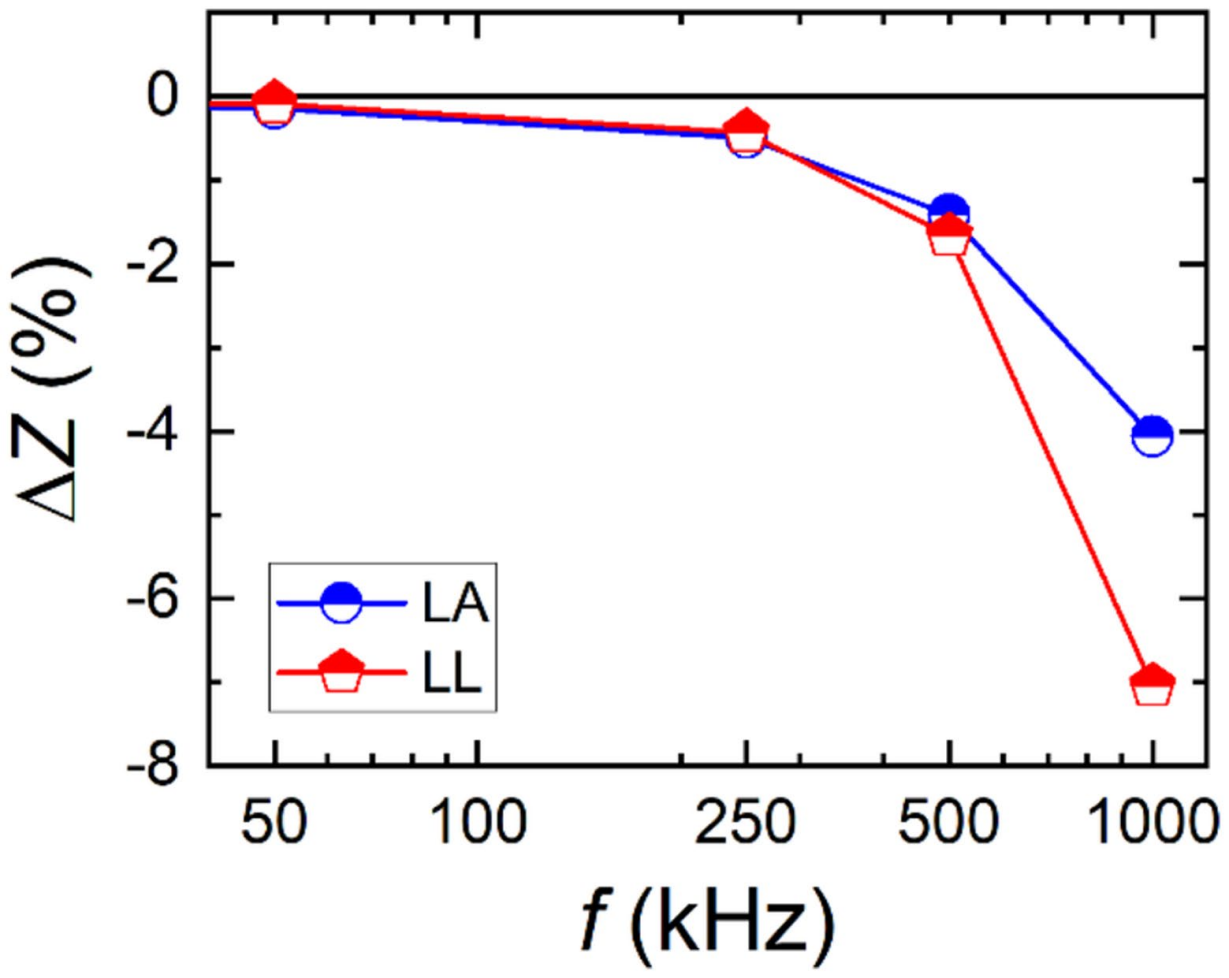
Relative changes of left arm (*Z*_LA_) and left leg (*Z*_LL_) impedance values with respect to the zero series resistor (no series resistor) conditions against the frequency. Series resistors of 100 Ω were attached at the current electrodes of left arm (LA) and left leg (LL) and at the potential electrodes of left arm (LA) and right arm (RA).

Among the measurement data, a case with 100 Ω resistors attached to both the current and the potential electrodes was selected to further examine the influence of contact resistance over the whole frequency range. Relative deviations in the measured impedance with respect to the zero series resistor condition were plotted against the frequency for *Z*_LA_ and *Z*_LL_ as shown in Figure 11. The impedance values deviated more as the measurement frequency increased; they decreased by more than 7% at 1 MHz. The larger deviation at the higher frequency might be attributed to the lower impedance contribution of the capacitance component as the capacitive impedance decreases with the increasing frequency.

**Figure 12:**
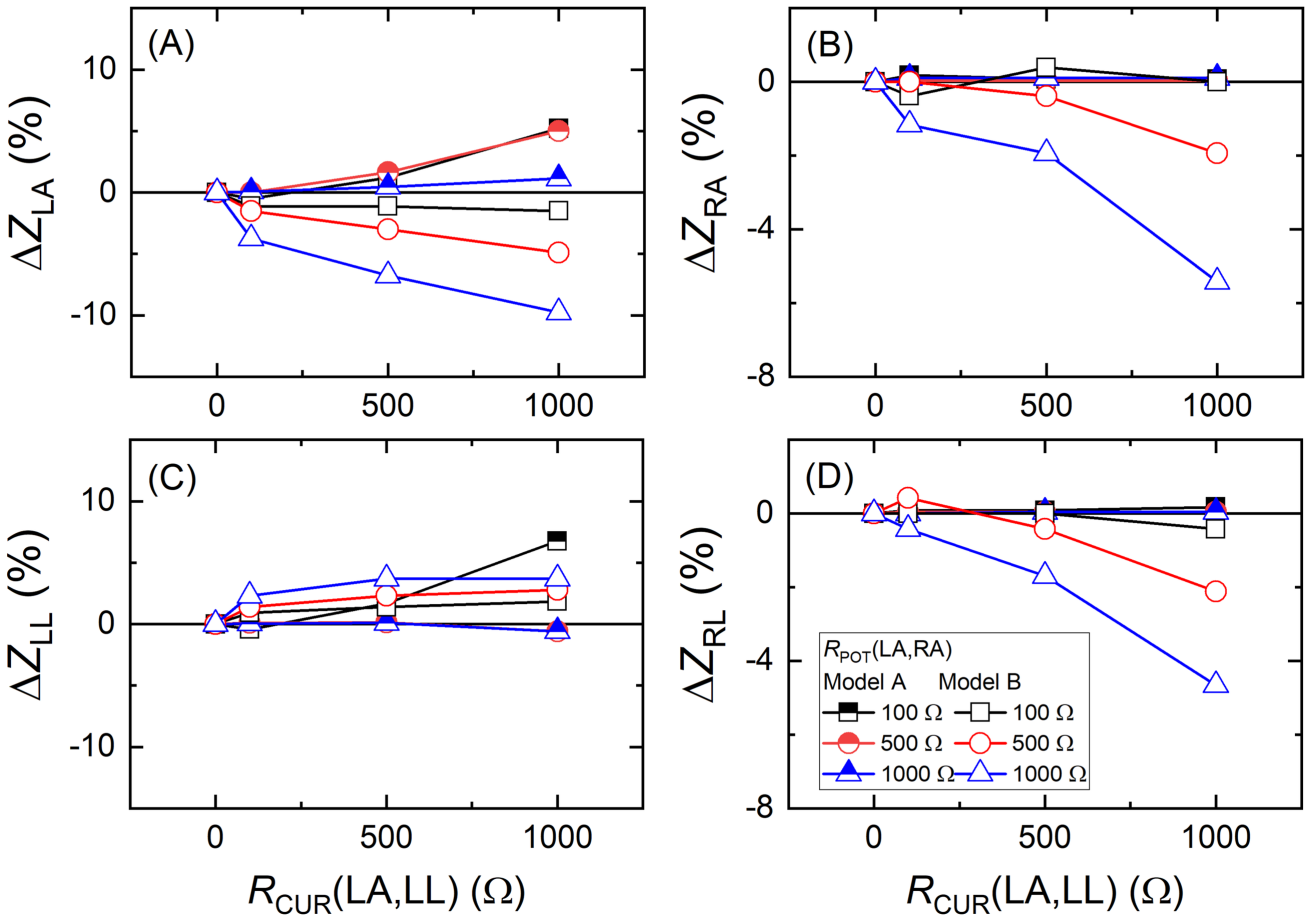
Relative changes of **(A)** left arm (*Z*_LA_), **(B)** right arm (*Z*_RA_), **(C)** left leg (*Z*_LL_), and **(D)** right leg (*Z*_RL_) impedance values with respect to the zero series resistor (no series resistor) conditions against the varying series resistances at 250 kHz for two different body impedance analysis devices of model A and B. The series resistors were attached at the potential electrodes of LA & RA and the current electrodes of LA & LL as shown in Figure 10A.

Furthermore, the effects of the contact resistance of the two BIA models of A and B were compared with series resistors at the electrodes. The results are presented in Figure 12, where relative changes in the measured impedance values at 250 kHz are plotted against the series resistance at the current electrodes of LA and LL for different series resistances at the potential electrodes of LA and RA. It was interesting to observe that two models showed rather opposite behaviors. For instance, it can be seen in Figure 12A that the measured *Z*_LA_ changed more in the model A when the resistance of the series resistors at the potential electrodes were smaller, while the opposite was observed in model B. Additionally, sign of the impedance change was reversed. On the other hand, for the right body parts of RA and RL, the measured impedance of model A changed slightly, but that of model B changed significantly. It seems like that the correlation in impedance measurements between body parts is relatively high for model B. Again, two BIA models reacted quite differently to the series resistor attachments.

In terms of the body composition results, analyzed based on the impedance measurements, the amount of body fat changed by 10% when the measured impedance was changed by 10% for both BIA models though sign of the change was opposite.

### 3.5 Temperature and humidity

Since the resistors and capacitors of the imitator are passive elements with temperature and humidity coefficients, the environmental effects were also evaluated. Thus, the imitator was placed in a chamber where both temperature and humidity can be controlled, and its impedance was measured using the BIA model A while varying the environmental condition. First, the temperature was varied from 10 °C to 40 °C with the humidity fixed at 50% RH. The impedance remained the same within 2% range for all body parts over the whole measurement frequency range despite the temperature variation. Next, the humidity was varied from 35 to 80% with the temperature fixed at 25 °C. Again, similar results were observed; negligible impedance changes within 1.5% range for the varying humidity levels. Thus, the stability of the BII was evaluated against the temperature and the humidity.

## 4 Body impedance analysis device calibration and discussions

**Figure 13:**
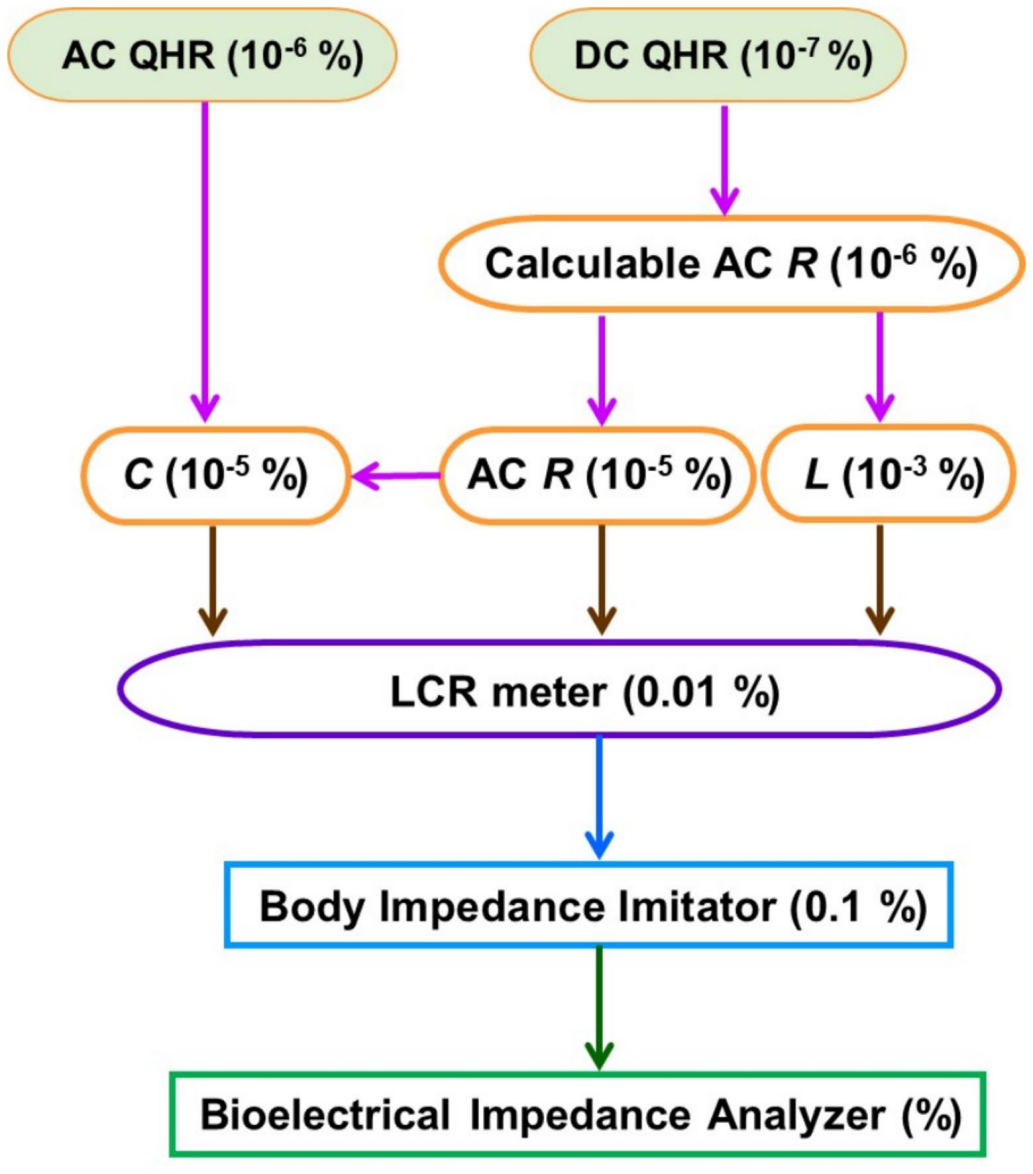
Calibration process for a body impedance analysis device using a body impedance imitator. The numbers in the parentheses represent the calibration uncertainty level of each impedance standards or meters.

Based on the previous investigations, a calibration process is proposed for a BIA device using a BII, as illustrated in Figure 13. A BIA device can be calibrated using a BII whose impedance is already calibrated using a reference LCR meter.

When calibrating the LCR meter, impedance standards with known reference values (capacitance *C*, AC resistance AC *R*, and inductance *L*) are employed, which are traceable to national resistance standards (AC or DC quantum Hall resistance) (39–41). For instance, calculable resistance standards, whose frequency dependencies can be calculated based on their geometric parameters, can be employed. Well-made calculable standards in the metrology grades exhibit AC values, which deviate only slightly from DC values; AC-DC difference levels of 10^−9^ near 1 kHz and 10^−4^ at 1 MHz (42).

**Figure 14:**
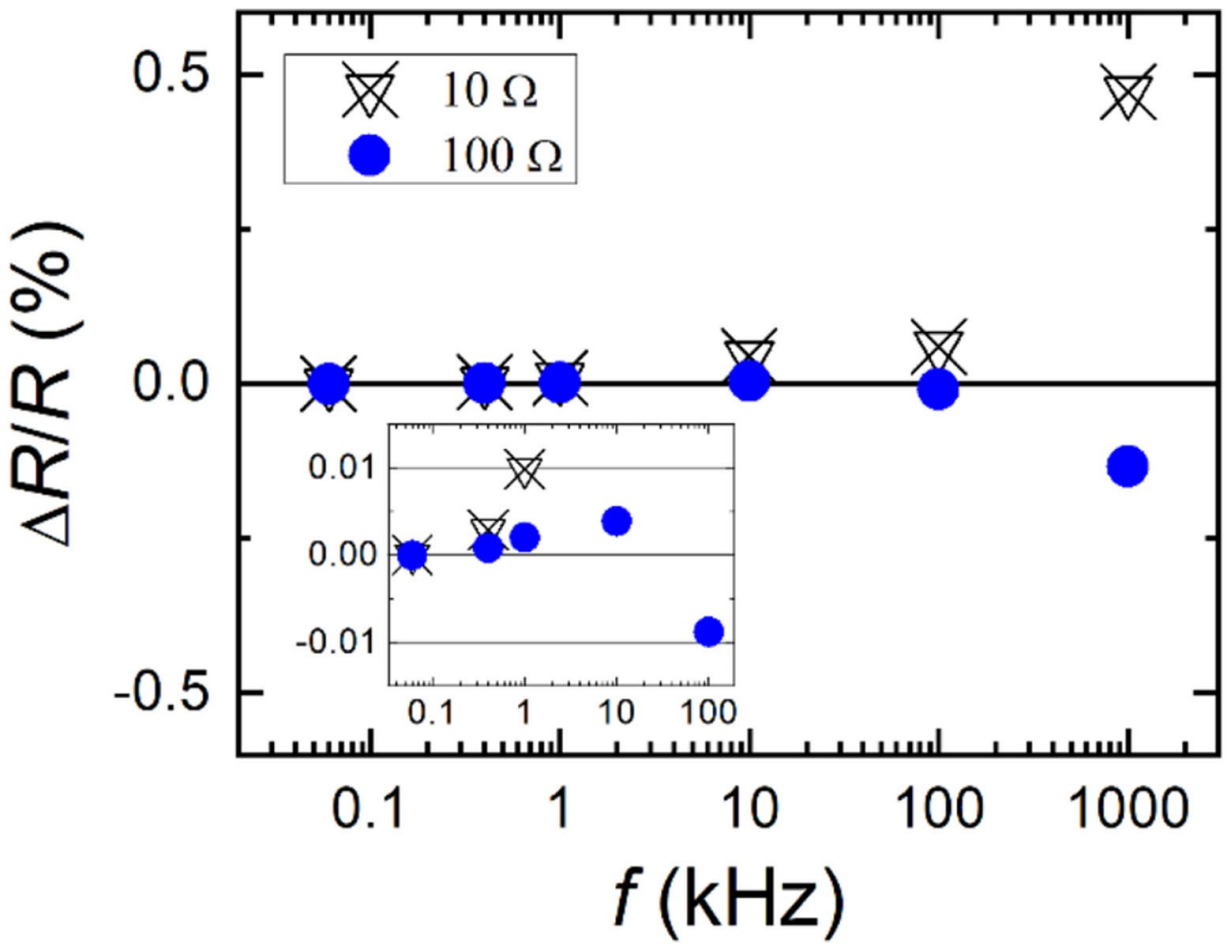
Relative changes of AC–DC calculable resistance values, measured by an LCR meter, against the frequency.

Typical LCR meters can measure the electrical impedance up to a few MHz, and their impedance measurement range well covers the BIA device measurement range of 10 Ω and 1,000 Ω. Figure 14 shows measurement results for calculable resistance standards of 10 Ω and 100 Ω using an LCR meter. Relative changes in the resistance values with respect to the initial frequency point of 60 Hz are plotted. As seen, some frequency dependency can be observed on level of 0.1% for 100 Ω and 0.5% for 10 Ω, respectively, at the highest frequency of 1 MHz. The inset shows that the change in the 100 Ω resistance stayed within 0.01% up to 100 kHz. It infers that the LCR meter is capable of measuring impedance values with a confidence level of 0.01% at the best. Hence, the developed BIIs were evaluated using the calibrated LCR meter. Through this unbroken chain of calibrations, a BIA device can be assessed based on the BII for its impedance measurement reliability.

Once the BII is calibrated using the reference LCR meter, it is measured by a BIA device to be calibrated. The impedance measurement uncertainty is given as well to present the confidence level of the measured data. There are several factors to be considered for a precise calibration: stabilization of both the BII and the BIA in the measurement environment and proper contact between the BII and the BIA electrodes.

The measurement uncertainty was analyzed as presented in Table 1 for the BIA device model A. There are two types of uncertainty factors. First, the type A uncertainty represents the measurement repeatability, obtained from a statistical variation calculation of the measurement data. On the other hand, the type B uncertainty factors originate from the BII, the BIA device, environment, etc.

**Table 1:** Calibration uncertainty of the body impedance analysis device model A using a body impedance imitator.

Uncertainty contribution	Type	RA/LA/RL/LL (%)	TR (%)
1. Imitator calibration	B	0.05	0.65
2. Imitator stability	B	0.00	0.00
3. BIA accuracy	B	0.32	0.40
4. BIA resolution	B	0.01	0.03
5. BIA linearity	B	0.43	0.58
6. Frequency	B	0.06	0.58
7. Temperature	B	0.40	0.58
8. Contact resistance	B	0.10	0.14
9. Connecting lead wire	B	0.00	0.00
10. Repeatability	A	0.01	0.00
*Combined standard uncertainty*		*0.7*	*1.3*
*Expanded uncertainty (k = 2)*		*1.4*	*2.6*

Since the BII is employed as a reference standard, its calibration uncertainty and stability contribute to the BIA device calibration uncertainty. The BII was calibrated using the reference LCR meter, and its stability was studied as both short- and long-term stabilities of the BII must be considered. The short-term stability, which can be affected by the environment factors or the measurement system noise, is already considered in the type A uncertainty. Whereas, the long-term stability needs to be taken into account if the BII is not used immediately after a calibration, because its calibration value may change over time.

The other type B uncertainty factors, arising from the BIA device itself are determined by instrument specifications along with measurement environmental factors such as temperature and connecting lead wires. Each uncertainty factor was carefully evaluated as mostly described in the previous section. As shown in Table 1, accuracy, linearity, and temperature are more contributing factors by about one order of magnitude when compared to other factors. After individually evaluating the uncertainty factors, all the factors were combined by a root sum square. As a result, the expanded uncertainty was calculated to be about 1.4% for the limbs and 2.6% for the trunk with a confidence level of 95% (*k* = 2).

## 5 Conclusion

Extensive studies have been performed on the multi-frequency BIA devices to explore the reliability of the measurement data. First of all, a BII was developed as a stable reference for the BIA device calibration, because a real human body cannot be a practical solution. The home-built BII was fabricated according to the body circuit model with five body parts of four limbs and one trunk. Each part consists of *R*_P_, *R*_S_, and *C*_S_, representing the extracellular fluid, the intracellular fluid, and the cell membrane, respectively. The impedance of each body part was designed to be adjustable in the range of 1 Ω to 1,000 Ω.

Using the developed BII, two BIA device models of A and B with similar specifications were evaluated in terms of measurement accuracy, resolution, electrode contact, etc. Both models measure the body impedance with eight-polar configuration at multiple frequencies between 1 kHz and 1 MHz. It was found that two BIA models, despite of their similar measurement principles and claimed capabilities, showed some significant differences in their measurement performance. Such distinction between the measurement data of the BIA devices emphasizes the motivation of this study, and the instrument calibration can ensure the confidence level of the measurement data.

Subsequently, a BIA device calibration process was suggested using the developed BII as a reference standard. The impedance values of the BII were calibrated in advance using an LCR meter, which also got calibrated based on impedance standards, traceable to national resistance standards. Afterwards, a BIA device was calibrated using the calibrated BII standard. Consequently, the BIA device measurement capability could be quantitatively evaluated. For instance, the BIA device model A was calibrated with the expanded uncertainty about 1.4% for the limbs and 2.6% for the trunk with a confidence level of 95% (*k* = 2).

In conclusion, BIA devices can be calibrated using the reference standards like BII for their impedance measurement capabilities, and their measurement data can be presented with a level of confidence, that is internationally accredited through a certified quality system. When manufacturers or users want to get their BIA devices calibrated, they can send their BIA devices or BII standards to accredited calibration laboratories. It would be easier to send the BII standard, and the calibrated BII standard can be used as a reference to evaluate the BIA device. The developed calibration procedure can be generally applied to BIA devices of eight-polar configuration. For other BIA devices of different configuration, it could be still employed after modifying the BII electrode configuration or connection accordingly.

## Data Availability

The original contributions presented in the study are included in the article/supplementary material, further inquiries can be directed to the corresponding authors.

## References

[1] Lukaski HC Johnson PE Bolonchuk WW Lykken GI. Assessment of fat-free mass using bioelectrical impedance measurements of the human body. Am J Clin Nutr. (1985) 41:810–7. doi: 10.1093/ajcn/41.4.810, 3984933

[2] Cornish BH Thomas BJ Ward LC. Improved prediction of extracellular and total body water using impedance loci generated by multiple frequency bioelectrical impedance analysis. Phys Med Biol. (1993) 38:337–46. doi: 10.1088/0031-9155/38/3/001, 8451277

[3] Foster KR Lukaski HC. Whole-body impedance: what does it measure? Am J Clin Nutr. (1996) 64:388S–96S. doi: 10.1093/ajcn/64.3.388S, 8780354

[4] Scharfettery H Hartingery P Hinghofer-Szalkayz H Hutteny H. A model of artefacts produced by stray capacitance during whole body or segmental bioimpedance spectroscopy. Physiol Meas. (1998) 19:247–61. doi: 10.1088/0967-3334/19/2/012, 9626689

[5] Ellis KJ Shypailo RJ Wong WW. Measurement of body water by multifrequency bioelectrical impedance spectroscopy in a multiethnic pediatric population. Am J Clin Nutr. (1990) 70:847–53. doi: 10.1093/ajcn/70.5.847, 10539745

[6] Bera TK. Bioelectrical impedance methods for noninvasive health monitoring: a review. J Med Eng. (2014) 2014:1–28. doi: 10.1155/2014/381251, 27006932 PMC4782691

[7] Kyle UG Bosaeus I De Lorenzo A Deurenberg P Elia M Gomez JM . Composition of the ESPEN working group. Bioelectrical impedance analysis—part I: review of principles and methods. Clin Nutr. (2004) 23:1226–43. doi: 10.1016/j.clnu.2004.06.004, 15380917

[8] Guida B Laccetti R Gerardi C Trio R Perrino NR Strazzullo P . Bioelectrical impedance analysis and age-related differences of body composition in the elderly. Nutr Metab Cardiovasc Dis. (2007) 17:175–80. doi: 10.1016/j.numecd.2005.11.001, 17367702

[9] Matthie JR. Bioimpedance measurements of human body composition: critical analysis and outlook. Expert Rev Med Devices. (2008) 5:239–61. doi: 10.1586/17434440.5.2.239, 18331184

[10] Yeh C Chen YJ Lai LY Jang TR Chiang J Chen YY . Bioelectrical impedance analysis in a mathematical model for estimating fat-free mass in multiple segments in elderly Taiwanese males. Int J Gerontol. (2012) 6:273–7. doi: 10.1016/j.ijge.2012.01.031

[11] Chinen K Kinjo I Zamami A Irei K Nagayama K. New equivalent-electrical circuit model and a practical measurement method for human body impedance. Biomed Mater Eng. (2015) 26:S779-86. doi: 10.3233/BME-151369, 26406074

[12] Brantlov S Jødal L Andersen RF Lange A Rittig S Ward LC. Bioimpedance resistance indices and cell membrane capacitance used to assess disease status and cell membrane integrity in children with nephrotic syndrome. Sci World J. (2019) 2019:1–8. doi: 10.1155/2019/4274856, 31210755 PMC6532278

[13] Nwosu AC Mayland CR Mason S Cox TF Varro A Stanley S . Bioelectrical impedance vector analysis (BIVA) as a method to compare body composition differences according to cancer stage and type. Clin Nutr ESPEN. (2019) 30:59–66. doi: 10.1016/j.clnesp.2019.02.006, 30904230

[14] Tinsley GM Harty PS Moore ML Grgic J Silva AM Sardinha LB. Changes in total and segmental bioelectrical resistance are correlated with whole-body and segmental changes in lean soft tissue following a resistance training intervention. J Int Soc Sports Nutr. (2019) 16:58. doi: 10.1186/s12970-019-0325-4, 31783760 PMC6883592

[15] Bracco D Thiébaud D Chioléro RL Landry M Burckhardt P Schutz Y. Segmental body composition assessed by bio-electrical impedance analysis and DEXA in humans. J Appl Physiol. (1996) 81:2580–7. doi: 10.1152/jappl.1996.81.6.2580, 9018509

[16] Bedogni G Malavolti M Severi S Poli M Mussi C Fantuzzi A . Accuracy of an eight-point tactile-electrode impedance method in the assessment of total body water. Eur J Clin Nutr. (2002) 56:1143–8. doi: 10.1038/sj.ejcn.1601466, 12428182

[17] Medici G Mussi C Fantuzzi A Malavolti M Albertazzi A Bedogni G. Accuracy of eight-polar bioelectrical impedance analysis for the assessment of total and appendicular body composition in peritoneal dialysis patients. Eur J Clin Nutr. (2005) 59:932–7. doi: 10.1038/sj.ejcn.1602165, 15928682

[18] Bosy-Westphal A Later W Hitze B Sato T Kossel E Glüer C . Accuracy of bioelectrical impedance consumer devices for measurement of body composition in comparison to whole body magnetic resonance imaging and dual X-ray absorptiometry. Obes Facts. (2008) 1:319–24. doi: 10.1159/000176061, 20054195 PMC6452160

[19] Ling CHY de Craen AJM Slagboom PE Gunn DA Stokkel MP Westendorp RGJ . Accuracy of direct segmental multi-frequency bioimpedance analysis in the assessment of total body and segmental body composition in middle-aged adult population. Clin Nutr. (2011) 30:610–5. doi: 10.1016/j.clnu.2011.04.001, 21555168

[20] Lee JB Sung BJ Ko BG Cho EH Seo TB. A comparative study on the reliability and validity of body composition results by impedance method measurement device. J Exerc Rehabil. (2023) 19:299–308. doi: 10.12965/jer.2346404.202, 37928832 PMC10622934

[21] Looney DP Schafer EA Chapman CL Pryor RR Potter AW Roberts BM . Reliability, biological variability, and accuracy of multi-frequency bioelectrical impedance analysis for measuring body composition components. Front Nutr. (2024) 11:1491931. doi: 10.3389/fnut.2024.1491931, 39691170 PMC11649400

[22] Oldham NM. Overview of bioelectrical impedance analyzers. Am J Clin Nutr. (1996) 64:405S–12S. doi: 10.1093/ajcn/64.3.405S, 8780356

[23] Freeborna TJ Milligana A Esco MR. Evaluation of ImpediMed SFB7 BIS device for low-impedance measurements. Measurement. (2018) 129:20–30. doi: 10.1016/j.measurement.2018.07.010

[24] Marcotuli V Zago M Moorhead AP Vespasiani M Vespasiani G Tarabini M. Metrological characterization of instruments for body impedance analysis. Acta IMEKO. (2022) 11:1–7. doi: 10.21014/acta_imeko.v11i3.1179

[25] Son JW Han BD Bennett JP Heymsfield S Lim S. Development and clinical application of bioelectrical impedance analysis method for body composition assessment. Obes Rev. (2025) 26:e13844. doi: 10.1111/obr.13844, 39350475

[26] Branco MG Mateus C Capelas ML Pimenta N Santos T Mäkitie A . Bioelectrical impedance analysis (BIA) for the assessment of body composition in oncology: a scoping review. Nutrients. (2023) 15:4792. doi: 10.3390/nu15224792, 38004186 PMC10675768

[27] Porta EL Faragli A Herrmann A Muzio FPL Estienne L Nigra SG . Bioimpedance analysis in CKD and HF patients: a critical review of benefits, limitations, and future directions. J Clin Med. (2024) 21:6502. doi: 10.3390/jcm13216502 PMC11546501 39518641

[28] Nwosu AC Stanley S Mayland CM Mason S McDougall A Ellershaw JE. Non-invasive technology to assess hydration status in advanced cancer to explore relationships between fluid status and symptoms: an observational study using bioelectrical impedance analysis. BMC Palliat Care. (2024) 23:209. doi: 10.1186/s12904-024-01542-z 39160544 PMC11331739

[29] Prior-Sánchez I Herrera-Martínez AD Zarco-Martín MT Fernández-Jiménez R Gonzalo-Marín M Muñoz-Garach A . Prognostic value of bioelectrical impedance analysis in head and neck cancer patients undergoing radiotherapy: a VALOR® study. Front Nutr. (2024) 11:01–13. doi: 10.3389/fnut.2024.1335052, 38463940 PMC10921554

[30] Abasi S Aggas JR Garayar-Leyva GG Walther BK Guiseppi-Elie A. Bioelectrical impedance spectroscopy for monitoring mammalian cells and tissues under different frequency domains: a review. ACS Meas Sci Au. (2022) 2:495–516. doi: 10.1021/acsmeasuresciau.2c00033, 36785772 PMC9886004

[31] Chumlea WC Guo SS. Bioelectrical impedance: a history, research issues, and recent consensus. Washington, DC: National Academies Press (US) (1997). 7:169.

[32] Brantlov S Ward LC Isidor S Hvas CL Rud CL Jødal L. Cell membrane capacitance (cm) measured by bioimpedance spectroscopy (BIS): a narrative review of its clinical relevance and biomarker potential. Sensors. (2025) 25:4362. doi: 10.3390/s25144362, 40732489 PMC12299080

[33] Deurenberg P Weststrate JA Paymans I van der Kooy K. Factors affecting bioelectrical impedance measurements in humans. Eur J Clin Nutr. (1988) 42:1017–22. doi: 10.1038/ejcn.1988.136 3234328

[34] Roos AN Westendorp RGJ Froehlich M. Tetrapolar body impedance is influenced by body posture and plasma sodium concentration. Eur J Clin Nutr. (1991) 46:53–60. doi: 10.1038/ejcn.1992.8 1559508

[35] Liang MT Norris S. Effects of skin blood flow and temperature on bioelectric impedance after exercise. Med Sci Sports Exerc. (1993) 25:1231–9. doi: 10.1249/00005768-199311000-00005, 8289609

[36] Di Iorio BR Terracciano V Bellizzi V. Bioelectrical impedance measurement: errors and artifacts. J Ren Nutr. (1999) 9:192–7. doi: 10.1016/S1051-2276(99)90033-X 10528051

[37] Slinde F Bark A Jansson J Rossander-Hultheln L. Bioelectrical impedance variation in healthy subjects during 12 h in the supine position. Clin Nutr. (2003) 22:153–7. doi: 10.1054/clnu.2002.0616, 12706132

[38] Dixon CB Ramos L Fitzgerald E Reppert D Andreacci JL. The effect of acute fluid consumption on measures of impedance and percent body fat estimated using segmental bioelectrical impedance analysis. Eur J Clin Nutr. (2009) 63:1115–22. doi: 10.1038/ejcn.2009.42, 19536161

[39] Kim DB Kassim DM Kim WS Callegaro L D'Elia V Trinchera B . Traceability chain at KRISS from DC quantum hall resistance to farad using coaxial bridges. IEEE Trans Instrum Meas. (2019) 68:1941. doi: 10.1109/TIM.2019.2896365

[40] Tran NTM Kucera J Kim WS Kim DB. Calibration of 10 nF capacitance standard from DC quantum hall resistance using a digital impedance bridge. Meas Sci Technol. (2023) 34:075009. doi: 10.1088/1361-6501/acc6e2

[41] Kim DB Shin SS Kim WS Kucera J. Realization of inductance scale using digital bridges at low frequencies. IEEE Trans Instrum Meas. (2024) 73:1005207. doi: 10.1109/TIM.2024.3364969

[42] Awan SA Kibble BP Schurr J. Coaxial electrical circuits for interference-free measurements. London: IET Electrical Measurement Series (2011).

